# Community Exercise: A New Tool for Personalized Parkinson’s Care or Just an Addition to Formal Care?

**DOI:** 10.3389/fnsys.2022.916237

**Published:** 2022-06-30

**Authors:** Josefa Domingos, John Dean, Júlio Belo Fernandes, João Massano, Catarina Godinho

**Affiliations:** ^1^Grupo de Patologia Médica, Nutrição e Exercício Clínico (PaMNEC) do Centro de Investigação Interdisciplinar Egas Moniz (CiiEM), Almada, Portugal; ^2^Department of Neurology, Donders Institute for Brain, Cognition and Behaviour, Radboud University Medical Center, Nijmegen, Netherlands; ^3^Triad Health AI, Aurora, CO, United States; ^4^Young Parkies, Porto, Portugal; ^5^Department of Neurology, Centro Hospitalar Universitário de São João, Porto, Portugal; ^6^Department of Clinical Neurosciences and Mental Health, Faculty of Medicine, University of Porto, Porto, Portugal

**Keywords:** Parkinson’s disease, community exercise programs, physiotherapy, exercise, dance, boxing, nordic walking, tai chi

## Abstract

Physiotherapy and exercise are associated with motor and non-motor benefits in Parkinson’s disease (PD). Community exercise programs may increase ongoing exercise participation and help people with Parkinson’s disease actively participate in their health management. But there is still limited knowledge about these programs regarding their benefits, safety, implications over the long-term, and effective implementation. These questions could hold relevant clinical implications. In this perspective article, we identify the current challenges and reflect upon potential solutions to help community exercise to be implemented as an additional anchor to personalize management models for Parkinson’s disease.

## Current Care for Parkinson’S Disease Introduction

Parkinson’s disease (PD) is characterized by complex motor and non-motor features, managed through pharmacological and non-pharmacological treatment options (Bloem et al., 2021). Non-pharmacological interventions such as physiotherapy, speech therapy, PD nurse specialist care, and occupational therapy help improve patients’ functioning and assist patients and their families to cope with disability (Clarke et al., 2006; Keus et al., 2014; Lennaerts et al., 2017; Radder et al., 2020). Physiotherapy is among the most studied non-pharmacological treatments and has shown benefits in improving motor impairments in transfers, posture, reaching and grasping, balance, freezing, falls, gait, and physical capacity. Exercise is often applied as part of physiotherapy interventions (Keus et al., 2014; Radder et al., 2020). There is growing evidence showing its effectiveness for several motor (Keus et al., 2014; van der Kolk et al., 2019; Radder et al., 2020) and non-motor problems (Cusso et al., 2016) but ongoing exercise is needed to maintain benefits (Keus et al., 2014).

**Figure 1:**
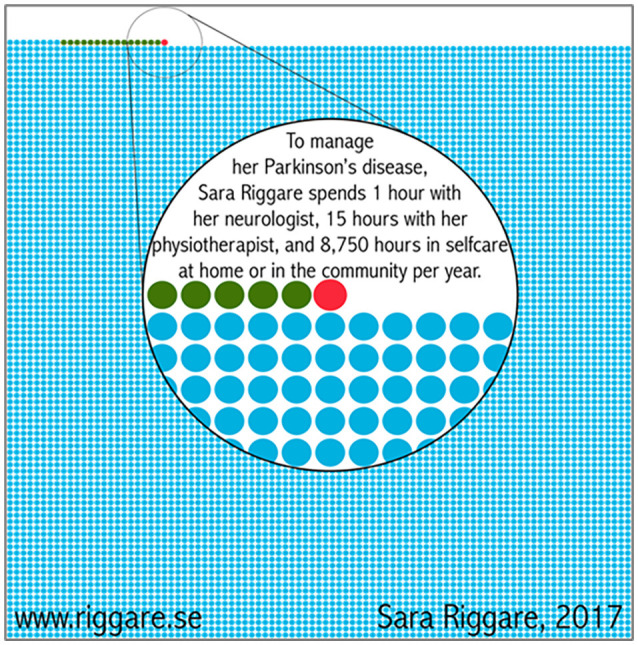
Patients’ perspective on common care from Sweden.

People with PD can receive this care in a variety of ways and in a range of settings such as inpatient, outpatient, home-based care, and community care. Still, current healthcare delivery is often complex, fragmented, and imposes numerous barriers (e.g., limited expertise, poor communication, geographical distances, financial asymmetries, lack of time, and overburdened care partners; Schootemeijer et al., 2020). Depending upon the healthcare system, a patient may see their neurologist once or twice a year, for approximately 30 min each session. They may have an additional 15 h with their physiotherapist within that timeframe. But during the rest of the year, patients spend their time in self-care at home or in the community (Riggare and Hagglund, 2018; Figure 1). Noticeably, a study that followed 187 people with PD, showed that after 20 years of diagnosis, 47% were doing physiotherapy and 54% were doing some form of exercise in the community (Hassan et al., 2015). This illustrates well that, even in the later stages of the disease, people still significantly depend on local community resources.

Offering exercise in the community to deliver accessible care close to patients’ homes can help overcome some of these barriers and may prove an attractive solution to enhance ongoing exercise. Yet, despite promising results, there is still limited data concerning the true benefits, including the implications of long-term or continuous use, as well as insufficient information regarding safety, delivery and implementation methods. Ultimately, should these programs be included in current care models *per se*, or should it be organized outside the care system, as an addition to formal care?

Therefore, in this viewpoint, we will reflect on key questions that may revolutionize PD care and fuel future research, including: Which community programs have been studied in PD? What are the benefits of these programs within current PD care models? What are the current drawbacks, limitations, and future needs to implement such community programs? Can technology be used to benefit access to these community programs? What initiatives/actions or possible solutions can be put in place to achieve more personalized community evidence-based care?

## The Use of Community Exercise Programs in Current Parkinson’S Disease Care Models

Community exercise programs commonly refer to the use of different exercises in a group format, delivered close to a person’s home, in community centers or local rehabilitation centers, and most frequently delivered by non-medical professionals. A wide range of Parkinson-specific community-based programs are already being used in PD. The most common ones outlined in recent literature include: dance (Kalyani et al., 2019), boxing (Combs et al., 2013; Domingos et al., 2019), Nordic walking (Granziera et al., 2021), Tai Chi (Liu et al., 2019), Qigong (Chen et al., 2020), and aquatic exercise (Perez-de la Cruz et al., 2016; Carroll et al., 2017; Kurt et al., 2018; Silva and Israel, 2019).

Dance is one of the most widely researched forms of exercise in PD and has been shown to have a beneficial effect on motor symptoms, balance, and gait (Dos Santos Delabary et al., 2018; Radder et al., 2020). It has also shown potential benefits on cognitive function, mood, and quality of life (Kalyani et al., 2019). Tai Chi and Qigong challenge primarily balance and are also recognized by international guidelines as intervention options to improve balance and gait impairments (Clarke et al., 2006; Keus et al., 2014). Tai Chi (Liu et al., 2019) and Qigong (Chen et al., 2020) compared with no exercise or sham treatment revealed a beneficial effect on motor symptoms, balance, and gait parameters. Nordic Walking (Granziera et al., 2021) when compared to no exercise or sham treatment, has also been shown to have a moderately large effect on motor symptoms and a large effect on balance (Berg Balance Scale) and gait (6-min walk test). Hydrotherapy interventions, in comparison with standard physiotherapy or no exercise, have shown a moderately large effect on the Timed Up and Go test and a moderately large effect was found for reducing the fear of falling (Radder et al., 2020). Boxing still has very limited evidence but is one of the most popular among people with PD (Domingos et al., 2019). In 2013 (Combs et al., 2013), the authors showed that boxing resulted in significant improvements in gait velocity and endurance over time, when compared to a conventional physiotherapy control group.

## The Benefits of Community Care for PD

Community programs are spreading globally as a practical and cost-effective way to assist people with PD living in the community to actively participate in their health management. These community programs are a critical exercise resource for people with PD for several reasons.

First, they offer a means of ongoing care that may help preserve the patient’s physical and mental wellbeing by promoting an active lifestyle and by enhancing long-term exercise adherence. Physiotherapy and exercise interventions can improve physical functioning (Keus et al., 2014; Radder et al., 2020) but, as mentioned previously, continuous exercise is needed to maintain results. Compliance with such prolonged programs remains a critical challenge in PD (Keus et al., 2014; Schootemeijer et al., 2020). One possible solution to promote a sustained adherence to exercise is through continuous participation in these community exercise programs.

Second, people with PD have reported that they are more willing to participate in exercise programs that cost less and involve less travel (Ypinga et al., 2018). Given that community exercises are often close to participant’s home, these programs improve accessibility, and reduce transportation needs in people already dealing with increased mobility limitations, cognitive challenges, and reduced driving capacity and also reduce care partner burden.

Third, if these programs are appropriately designed and delivered, they can have a powerful impact on clinical outcomes and improve patient overall satisfaction with healthcare (Ypinga et al., 2018). For example, community programs such as dance or Tai Chi have been shown to potentially reduce fall risk factors such as balance, gait, and cognition (Kalyani et al., 2019). As a result, their usefulness to society increases tremendously given the physical and mental impact they may have on preventing falls.

Fourth, health care systems benefit from cost savings achieved from community care. Policy makers throughout the world are keen to implement and spread low-cost models to improve the quality of care (Bloem et al., 2017). Ultimately, favoring the use of community exercise programs with improved quality and safety can complement current healthcare options in a more beneficial manner but also help to ascertain an affordable healthcare.

## Current Challenges in Community Care (And Potential Solutions)

We identify several challenges that can slow the implementation of quality care at four different levels: research, instructor, organizational, and patient.

### Research Level

At the research level, the extent to which general exercise programs in the community can be translated into sustainable, adequate, and safe exercise programs specific to PD is still unclear. There is limited evidence on the optimal design, delivery, and implementation of PD-specific exercise programs that are easily accessible and generalizable to a large proportion of people with PD. Having more funding opportunities for studying non-pharmacological interventions is critical to generate further research, properly designed, sufficiently powered, and with proper regard for safety issues. Several questions should fuel this future research, including: Which type of exercises do people with PD prefer (and why)? Exactly what types of benefits do different exercises provide to specific phenotypes or stages of PD? What is the potential impact of demographics and subpopulation characteristics such as age, gender. and educational level, and disease-specific disabilities on the preference for this type of programs? Do program and participant characteristics influence people’s willingness to participate in these exercise programs? Given the overall financial costs, safety issues, and progressive nature of PD, which professionals should develop and who should deliver these community programs? Is there a need for added expertise, training, or clinical education for those applying for these programs in order to guarantee safety issues? With mobility difficulties and encroaching disability, how can technology support access to online community expert care? Should we wait for more robust evidence to implement these programs?

Importantly, although several studies have been conducted on the feasibility of some programs (Combs et al., 2013), there is virtually no satisfactory evidence to guide the implementation of specific evidence-based practices with clarity. Recommendations on how to adapt research exercise protocols into applicable to a “real-world” community setting, integrating the complexity of context and people participating, are urgently needed to inform future implementation efforts. Considerations need to be made for differences among certain disease severities, PD phenotype subpopulations, individuals who have cognitive impairment and practice setting (for example, rural as opposed to urban settings). People with PD are typically motivated to take part in research studies but following participation in an exercise intervention study, there is a general decline in activity levels. Research has shown that in order to maintain ongoing activity and keep physically active beyond the research setting, people with PD want evidence supporting the benefits of exercise as well as greater availability of programs closer to home, and guidance from health care providers toward exercise studies (Valadas et al., 2011). People with PD also considered that these exercise programs should consist of activities that are enjoyable, safe, and adaptable to the abilities of the individual, as well as include social engagement and social support (Zaman et al., 2021).

### Instructor Level

There is a clear need to enhance expertise among professionals delivering exercise programs to people with PD. International Physiotherapy guidelines advise patients to participate in the ongoing unsupervised exercise in their communities while also recommending people with PD to access expert care (Keus et al., 2014). Notably, there is an optimal minimal level of disease-specific expertise necessary, particularly with respect to the safe integration of exercises and reducing the risk of falls and other possibly dangerous issues (Keus et al., 2014). For example, being aware that cardiorespiratory regulation during exercise can be altered in people with PD when compared to age-matched controls (Sabino-Carvalho et al., 2018; Sabino-Carvalho and Vianna, 2020). The autonomic dysfunction can lead to inadequate hemodynamic responses, the failure to match the metabolic demands of working skeletal muscle, and exercise intolerance (Sabino-Carvalho et al., 2021). Better knowledge and understanding of these responses during exercise in this population is critical to guarantee safety during exercise. This means there is a need to be able to refer patients to a supportive and safe exercise environment that is preferably led by professionals with relevant Parkinson-specific training. By referring people with PD to attend exercise programs without expertise, health professionals could ultimately put the person’s safety at risk (Nijkrake et al., 2009; Keus et al., 2014). Importantly, people with PD have perceived the lack of PD expertise in community care programs as a primary area of weakness. Many individuals with PD express a strong interest in accessing this type of expertise within their community (Hirsch, 2009). Experience of working with the PD population is a significant source of expertise in and of itself; Nijkrake and colleagues have noted that physiotherapists with an annual treatment volume of at least seven people with PD report higher self-perceived expertise (Nijkrake et al., 2009; Ypinga et al., 2018). Presumably, this level of contact will not be difficult for many community exercise programs to achieve. Nonetheless, an effective educational component will be needed to complement this experiential expertise and bridge any gaps along the continuum of care. It is incumbent upon community exercise instructors to recognize limitations in their knowledge base, improve their ability to instruct and modify exercise approaches appropriately to maximize benefits, and to closely review their clinical needs in conjunction with their personal preferences in order to produce long-term benefits (Hirsch, 2009). Also, the instructors’ skills to identify good evidence from flawed evidence is essential given the current proliferation of scientific articles on exercise and PD. The provision of high-quality care is best achieved through evidence-based practice (Emparanza et al., 2015; NICE, 2015; Dean et al., 2021) and instructors should too practice in an evidence-informed manner. In addition, these instructors should seek additional guidance from other professionals when needed (Keus et al., 2014). Specific training, adequate ongoing educational support, and continuous contact with people with PD will be needed to practice in a manner that is supported by the emerging evidence (Nijkrake et al., 2009).

Additionally, to enhance accessibility to expertise, the use of technology specifically designed for use by people with PD may have an enormous role. Providing a co-approach combining technology and local in-person care community-based programs, which could be easily implemented in senior centers, is an intuitive example of how the use of technology can allow for access to expertise worldwide and ultimately may shape the near future. It would allow for more patients with PD to take part in such programs and also access PD expert care. Because technology raises concerns regarding usability and compliance in the more elderly population, having community centers organize spaces with technology and human support could significantly improve its successful implementation. The presence of a person for technical support represents a unique ability to provide a safe, facilitated, and remote intervention.

### Organizational Level

At the organizational level, among others, there is a lack of clear strategies to facilitate the quick dissemination of novel evidence-based practices into an ever-increasing number of unmonitored community programs. Clear leadership at the organizational level would help maintain patients’ trust in clinical practice and long-term adherence. An organized infrastructure could provide support to exercise instructors and other implementers to introduce updated practices and embrace additional changes whenever new evidence emerges. Additionally, raising awareness about these programs increases the public interest in these exercise programs, which will in turn influence the course of clinical research and consequently develop clinical practice. In this regard, patients’ associations could be excellent advocates, both informing and stimulating patients and their families.

To facilitate people with PD access to such programs society needs to build solutions for critical barriers impacting participation, including financial, traveling, physical and cultural barriers (Schootemeijer et al., 2020; Domingos et al., 2022). Evidence-based practices should be covered as a health insurance benefit. Health systems can implement new care models that shift funding using a mix of nonprofit and volunteer-run initiatives or integrated as part of the healthcare systems. Community initiatives (e.g., developing the concept of an exercise bus; creating safe places for exercise and walking paths that are accessible for parking) to support transportation can be put in place to reduce challenges in access. Technology is another way to facilitate access to specialized care and favor long adherence (Speelman et al., 2014). Several reports have shown the benefits of telemonitoring and telerehabilitation as a means to provide specialized care to patients who have difficulty accessing it, particularly during the coronavirus disease 2019 (COVID-19) pandemic (Helmich and Bloem, 2020; Domingos et al., 2022). Future studies may look to the potential value of applying technology to facilitate patient access to telemonitoring and specialized community-based programs in their homes (Speelman et al., 2014) or in community centers with remote trainers (Achey et al., 2014). Importantly, online programs should always include teaching courses on how to use technology to bypass limitations in its use by elderly populations.

### Patient Level

At the patient level, we will need to find solutions to bypass the several factors that may interfere with the ability of patients to participate, such as: fluctuations (motor symptoms or other) from day to day, concerns about becoming injured or falling, lacking sufficient time to exercise regularly, and perceive social stigma when exercising in public (Schootemeijer et al., 2020).

We also need to improve the awareness of people with PD with regard to evidence-based exercise programs and where such programs can be received. Society counts on the active participation of informed people with PD to make the right decisions for their health, capable of working as partners with all professionals (medical and non-medical) to optimize results (van der Kolk et al., 2019; Domingos et al., 2022). Yet, given the recent proliferation of exercise programs (with or without evidence), people with PD are faced with the challenge of how to choose. Making sense of these programs may also be complicated by the tendency among popular media to promote research results without consideration of the quality of the study design or the expertise of the research team. As a result, certain media sources might highlight a new exercise approach or other intervention that is unproven, and which may go on to run the risk of failing to produce the desired outcomes. For people with PD, this may result in disappointment and blunting of enthusiasm for participation in other interventions that could be more beneficial or more directly applicable to their specific deficits. In addition, financial resources utilized in the pursuit of less effective or possibly unhelpful exercise approaches will not be available for approaches with a proven track record of benefit.

Due to the complexity of these many factors, people with PD should count on health professionals and clinicians to advise them on trustworthy sources of information and options on care. Some guidance can also be achieved through awareness campaigns, research-based guidance programs (Domingos et al., 2021), or patient helplines. When providing care, health or other non-health professionals should use safe evidence-based practices. Such programs must be made available and accessible to people with PD all-inclusive to promote better care for people with PD, improve wellbeing, and ultimately reduce costs to health care systems. Additionally, care should be provided by professionals with PD expertise for better outcomes, reducing the use of unnecessary procedures and unrealistic expectations in people with PD, particularly for those with less favorable profiles for certain exercises.

We might be limited to understand the many heterogeneous factors that can interplay, several courses of action may already be possible to address the gap between expertise, evidence, dissemination, and implementation into community exercise practices. We summarize these potential actions discussed above in Table 1.

**Table 1:** Summary of potential courses of action needed to improve quality community care.

**Levels**	**Current challenge**	**Potential actions/measures**	**Overall aim**
Research level	Limited evidence for benefits and effective assessment measures	Continue to build specific evidence for different disease severity, PD phenotype subpopulations, among persons who have cognitive impairment, and in all practice settings.	Generating evidence to inform best practice
		Develop research with proper design, sufficient power and a proper regard for safety issues.	
		Make available more funding opportunities for non-pharmcological interventions	
	Limited translation of the research evidence to practice	Define recommendations on how to adapt research studies into practice. Use trained clinical Parkinson experts to deliver interventions in experimental studies consistently, with little variation across practices, and in accordance with guideline recommendations or research protocols	Refine the care strategy and its implementation. Inform future implementation efforts
Instructor level	Limited expertise among professionals delivering the programs	Support PD expertise among instructors *via* specific training, adequate ongoing educational support, and continuous contact with people with PD (by increasing referrals for increased case load and thus, more expertise)	Providing safe evidenced-based practices (better care).
			Reduce unnecessary procedures
			Reduce unrealistic expectations in people with PD, particularly for those with a less favorable profiles for certain exercises
Patient level	Barriers to participation in exercise	Implement strategies to bypass common barriers such as fluctuations in health, concerns about safety, time management strategies, culturally appropriate care, insufficiently engaging exercise options, use of technology to facilitate access to expert care.	Increase participation *via* easy access to safe, cost free, engaging exercise oportunities
		Provide programs that cost less, involve less travel, provide physical or psychological benefits and supervised by qualified professionals	
	Reduced awareness of such programs as options of care	Inform about access to these programs through awareness campagnes for people with PD regarding the existence evidence-based exercise programs and where such programs can be received	To generate informed people with PD that have active participation to make the right decisions for their health
Organizational level	Slow dissemination of the new evidence-based practices	Create an organized infrastructure to provide support to exercise instructors and other implementers to introduce consistently changing practices and embrace additional changes when never new evidence emerges.	Facilitate quick efficient dissemination of the new evidence-based practices.
		Use online communities to disseminate new research findings	Developing an evidence standard infrastructure for assuring and assessing the implementation of community practice
	Access to programs in the community	Reduce PD-specific critical barriers to participation: • 1-Traveling barriers with organized community transportation systems (e.g., “exercise bus”). Delivering online services with teaching courses on how to use technology. • 2-Financial barriers *via* provision of affordable services with covered services either through nonprofit, volunteer-run initiatives or integrated as part of the healthcare systems. Include a community exercise program as a health insurance benefit. • 3-Location barriers *via* creating safe places to exercise. • 4-Cultural barriers *via* hiring translators to favor underserved populations.	Making programs available and accessible to people with PD to promote better care and ultimately reduce costs to health care systems
	Reduced public awareness of existing programs in the community	Improve public awareness of how to access most beneficial exercise programs. Role for patient’s associations.	Raising awareness of evidence-based exercise programs to increased public interest in community exercise programs (and influence the direction of clinical research, and thus advance clinical practice)
	Unmonitored proliferation of programs	Redefine new care strategy for health system, definingclear desired outcomes and penalties for imposing practices upon people with PD that are not sufficiently evidence-based, including financial support and limited resources	Guarantee safe effective programs delivered

## Conclusions

Community exercise programs are growing in popularity and in research interest. These new initiatives increase optimism about the possibility of developing programs that are cost-effective, easily accessible and a means of providing structured, ongoing exercise programs for people with PD. Some changes in thinking by the healthcare community are needed to delineate how community services can better complement current clinical care and to prepare for their integration. These changes require shifting views, changing behaviors, incentives, and capacities to support this change, and developing novel models that allow these two areas to interact and coordinate effectively.

## Data Availability Statement

The original contributions presented in the study are included in the article, further inquiries can be directed to the corresponding author.

## Author Contributions

JDo: conceptualization, writing—original draft preparation. JDe: writing—original draft preparation, writing—review and editing. JF: writing—review and editing, supervision. JM: writing—review and editing. CG: conceptualization, writing—review and editing, supervision. All authors contributed to the article and approved the submitted version.

## Conflict of Interest

The authors declare that the research was conducted in the absence of any commercial or financial relationships that could be construed as a potential conflict of interest.

## Publisher’s Note

All claims expressed in this article are solely those of the authors and do not necessarily represent those of their affiliated organizations, or those of the publisher, the editors and the reviewers. Any product that may be evaluated in this article, or claim that may be made by its manufacturer, is not guaranteed or endorsed by the publisher.
